# Effect of Gamification on Task Engagement During an Eye-Tracking Test Battery in 5-Year-Old Children Born Preterm: Observational Study

**DOI:** 10.2196/77109

**Published:** 2025-10-27

**Authors:** Andrea De Gobbis, Marta Malavolta, Nathalie De Beukelaer, Els Ortibus, Aleksander Sadikov, Vida Groznik

**Affiliations:** 1 NEUS Diagnostics d.o.o. Ljubljana Slovenia; 2 Faculty of Computer and Information Science University of Ljubljana Ljubljana Slovenia; 3 Department of Development and Regeneration KU Leuven Leuven Belgium; 4 Leuven Child and Youth Institute KU Leuven Leuven Belgium; 5 Centre for Developmental Disabilities Universitair Ziekenhuis Leuven Leuven Belgium

**Keywords:** child, data accuracy, eye movement data analysis, eye-tracking, gamification, premature birth

## Abstract

**Background:**

Recent studies suggest that eye movements during tasks reflect cognitive processes and that analysis of eye movements using eye-tracking devices can identify developmental impairments in young children. Maintaining engagement during eye-tracking assessments in young children is challenging and often results in data loss due to distractions. This leads to incomplete recordings and repeated measurements, which can be taxing for young children. Gamification of eye-tracking procedures for cognitive diagnosis might increase engagement and help mitigate these problems, but its effects should be studied and quantified.

**Objective:**

This study compares a standard eye-tracking test battery designed by us with a gamified cartoon version to evaluate the effectiveness of gamification in reducing data loss. The gamified test incorporated child-friendly visuals to provide context for the stimuli presented. The study has two objectives: (1) to compare the data quality between the two versions of the test and (2) to investigate whether, by applying a dynamic stopping criterion to both tests, the higher data quality of the gamified procedure allows earlier test termination.

**Methods:**

Data were collected in a cohort of 25 children born preterm aged 5 years. We measured data quality using a metric derived from robustness, which we defined as the lost data index (LDI), along with task completion rates and feedback from participants. Data analyses were performed as follows: (1) direct comparison of the LDI for the two tests and (2) demonstrating that, although the base gamified test is longer, applying a stopping criterion results in comparable durations. The stopping criterion was based on the number of tasks with an LDI value below a predefined threshold.

**Results:**

The gamified version demonstrated a significant reduction in average LDI compared with the standard version in the first (*P*<.001, Mann-Whitney *U* test) and second (*P*=.01, *U* test) quarters of the test. In addition, a lower rate of missing values, concentrated at the beginning of the tests, allowed the cartoon test to be stopped after fewer tasks. This, together with the longer tasks of the cartoon test, resulted in comparable test lengths for all thresholds measured by area under the curve (*P*=.50, *U* test) and at the chosen threshold of 0.2 LDI (*P*=.21, *U* test). Increased engagement was further supported by positive feedback, with 79% (11/14) of the participants who provided feedback preferring the gamified version.

**Conclusions:**

These findings highlight the potential of serious games in eye-tracking–based cognitive assessments for 5-year-old children born preterm. Specifically, gamification might reduce missing values and increase participant engagement, leading to higher retention rates and more effective tests, without significantly lengthening testing procedures.

## Introduction

### Overview

Prematurity leads to an increased risk of altered neurodevelopmental outcomes in childhood and adolescence, such as cerebral palsy and impaired cognitive outcomes [1], often resulting from brain lesions [2], with many survivors facing a lifetime of disability [3]. Early diagnosis of these adverse outcomes is an important strategy, as it can lead to prompt treatment and a wider array of therapeutic options [2]. In this context, evidence suggests that oculomotor movements during specific tasks, such as smooth pursuit [4], reaction to stimuli [5], or scene exploration [6], serve as biomarkers of cognitive processes and show differences between children with cognitive impairments and typically developing children [7] that could be used to predict future development [8,9]. Therefore, a computerized neuropsychological test battery based on eye-tracking represents a noninvasive, qualitative assessment for the early detection of cognitive delays in children born preterm. Moreover, young nonverbal children are particularly suited to the eye-tracking approach, making it a desirable tool for early detection [10]. However, one of the main challenges when assessing younger participants is maintaining their engagement [11-13]. In a previous study [14], we suggested that certain paradigms for eye-tracking testing might be uninteresting to children born preterm aged 2-6 years.

Serious games offer a solution to this problem by incorporating dynamic and visually stimulating elements [15]. A gamified procedure reduces the monotony associated with repeated exposure to similar tasks, has been shown to lead to more consistent measurements [16], and can be administered remotely [17]. These characteristics are particularly important in pediatric assessments, where maintaining engagement is critical for data reliability [13]. However, the application of gamification to an eye-tracking paradigm introduces specific challenges, such as the possible addition of distracting elements [18-20]. Therefore, when introducing a gamified version of an existing cognitive assessment tool based on eye-tracking, it is necessary to directly compare the two versions [16,21].

### Related Works

#### Eye-Tracking

Different eye-tracking paradigms have been used to obtain measures related to visuocognitive functions in children, such as memory [22], social orienting [6], and smooth pursuit ability [4,23]. Measures include reaction times and looking times for visual processing [5,24,25], reaction times for processing speed and inhibition [26], gain, time delay, and error in smooth pursuit [4,23], and looking times for memory and social orienting [6,22]. In a recent study, Hokkenet et al [7] showed that the scan paths of children born preterm with cerebral visual impairment differ measurably from those of typically developing children.

The prevalence of missing values when testing young participants has been identified as one of the main challenges when using a remote eye-tracking system [12,27], which is an eye-tracking device fixed to a computer screen. Gredebäck et al [12] and Corbetta et al [11] suggested using engaging stimuli in the test to keep the attention of the child focused on the screen.

In adult and older adult populations, test batteries that combine different tasks have been used for the early diagnosis of dementia [28,29]. Such methodologies, which combine various kinds of stimuli and measure a variety of cognitive functions, provided a blueprint for our combined test battery. The potential carryover effects when combining multiple tasks in younger populations remain unclear, but the positive results obtained in adults [28,29] suggest that these types of tests provide a promising framework.

#### Gamification

The idea of using serious games to increase participant engagement in clinical tests is not new [15,18,30-32], and guidelines on introducing gamified elements to existing tests are available [33]. In their review, Lumsden et al [15] highlighted that serious games are often used to improve motivation and enhance appeal for a particular age group (either older or younger participants) during cognitive assessments. Conversely, other studies highlight that gamification might increase cognitive loads in adult participants [34], possibly introduce distracting elements that fatigue participants earlier [19,20], and may not influence data quality [35]. Another consideration when substituting a set of stimuli with different ones is the potential effect on measured outcomes [15]. For example, Kooiker et al [24] reported different reaction times to an animated cartoon stimulus versus a static shape. Held et al [19] hypothesized that gamification elements can distract participants from the core task, a view supported by Wiley et al [20], who suggest that gamification might introduce an expectation of an interesting activity that is later unmet. Therefore, when gamifying an existing paradigm, the adapted test may not be directly comparable with the original test. Even considering these drawbacks, in some cases presenting gamified stimuli may be necessary to keep young participants engaged, as they might otherwise get bored with the test. For example, the standard test we designed in a previous work [36] might have been too simplistic for children older than 24 months [14], leading to reduced engagement and data quality in a preliminary trial. Moreover, Wiley et al [16] reported that using game elements might even increase internal consistency and test-retest reliability.

#### Measuring Engagement

Improvements in eye-tracking technologies have allowed quantitative measurements of participant engagement [37,38]. Different methods can be used to assess engagement during testing from eye-tracking data, such as clustering of gaze trajectories [37] and inter-subject correlation [39], in conjunction with looking time and feedback. Finally, missing values are also useful for determining data quality [27,40], providing a baseline for the amount of usable data and offering an indication of participants’ engagement by measuring looking time at the screen.

Direct comparisons between two variants (ie, simple vs gamified) of the same cognitive assessment test to measure engagement and data quality using eye-tracking have been conducted previously in adult populations, with contrasting results. Scharingen et al [34], for example, suggested that gamification might increase cognitive load without strong evidence of improved attention, corroborating their findings with both eye-tracking measures and EEGs. In contrast, Friehs et al [21] reported that gamification can be beneficial for certain tasks, focusing on reaction times measured by an eye-tracking device, finding no differences in reaction times between versions and an increase in positive feedback with the gamified version. These conflicting findings underline the difficulties of directly comparing different procedures and measuring engagement and interest. A similar study by Gallagher et al [18] investigated the effects of gamification on children aged 8-12 years with attention deficit hyperactivity disorder, finding that game elements can increase intrinsic motivation but also distract participants from the task. However, the magnitude of the increase in intrinsic motivation was unclear because the authors did not directly compare the two versions of the task. To our knowledge, similar comparison studies have not been conducted in a sample of 5-year-old children born preterm. Therefore, we plan to address this research gap in this study by focusing on measures readily available during an eye-tracking experiment.

### This Study

The aim of this study is to compare data quality and engagement in a cohort of 5-year-old children born preterm for two variants of an eye-tracking test battery: a standard version [14,36] and a gamified cartoon version. The comparison is independent of task performance and uses, as a comparison measure, an index of lost data inspired by robustness [27,40], representing the ratio of nonmissing gaze points to the total number of eye-tracking samples. We propose that the resulting lost data index (LDI) not only provides an indication of data quality but also reflects test engagement by quantifying the time the participant spends looking at the screen, making it an optimal comparison metric. A secondary objective of this study is to show that, while gamification might increase the base duration of a test, using a dynamic stopping criterion based on data quality results in comparable test lengths for the two variants.

## Methods

### Overview

In this observational cohort study, we conducted a controlled experiment on a sample of 5-year-old children born preterm. The experimental procedure involved presenting each participant with two variants of the test battery, one after the other, in a pseudorandomized order.

**Figure 1:**
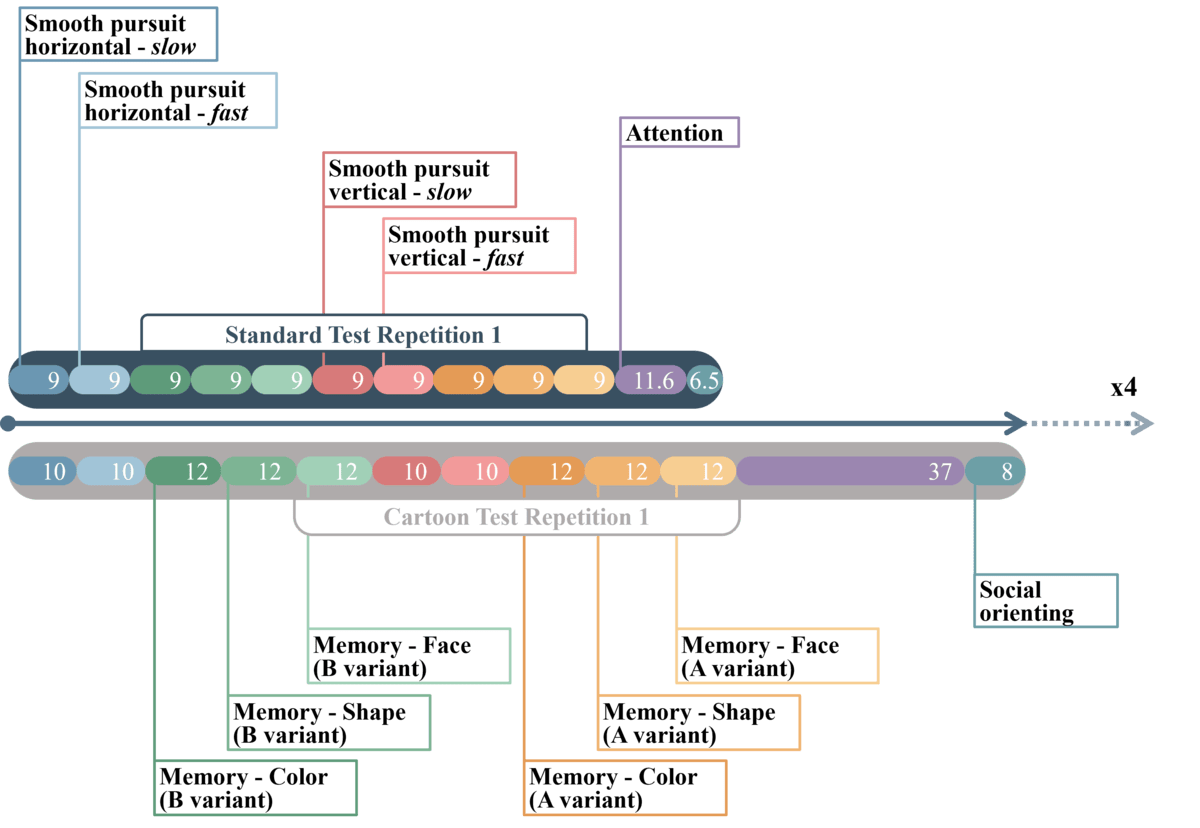
Basic structure of the two tests. Both tests are composed of 4 repetitions of the same 12 tasks. Task types follow the same order in both tests. Along with the task names, the duration of each task is indicated in seconds.

Both the standard and the cartoon test consist of a total of 12 tasks, including 4 smooth pursuit tasks, 6 memory tasks, 1 attention task, and 1 social orienting task. During each test, these tasks are presented in a fixed order (Figure 1) and repeated 4 times, for a total of 48 tasks comprising the complete test. The task order was chosen to maintain a sense of variety, with tasks based on scene exploration (memory task and social orienting task) alternated with more dynamic tasks (smooth pursuit task and attention task). The standard test builds upon the paradigm we introduced in previous works; for further details, we refer to De Gobbis et al [36] and Malavolta et al [14].

### The Standard and Cartoon Tests

The standard test is an evolution of an eye-tracking–based paradigm we developed in a previous study [14,36] and is composed of 4 types of tasks. Each task starts with the appearance of a yellow smiley face at the center of the screen for 1.5 seconds, accompanied by a sound, to focus the participant’s attention, and all stimuli are presented on a black background. The cartoon version was created to address a problem in the standard test identified in previous work [14]: participants aged 2-8 years tended to interrupt the test by standing up or becoming distracted. This lack of engagement suggested that the standard test was insufficiently interesting for this age group. To address this, we redesigned the test using the Unity game engine, incorporating dynamic cartoon elements to create a more engaging experience while preserving the core elements of the task.

To create the cartoon version of the test, we followed the RECIPE guidelines described by Nicholson [33]. Specifically, we aimed to create a context for the stimuli appearing on the screen by translating them into a cartoon setting. To make the comparison as fair as possible between the two versions, we did not introduce any interactive elements in the cartoon version and, similarly, no feedback was given on task performance. As such, we focused on what Nicholson defines as exposition and information, and what other authors refer to as intrinsic motivation [41], which, in short, consists of communicating to the participant the context of the action they are performing without a direct feedback loop, such as points. During the design of this gamified cartoon version, we focused on the following priorities: context and storytelling, visual interest, audio, and fidelity-engagement compromise.

- Context and storytelling: A narrative setting was introduced to provide context for the tasks, making them more engaging for participants. For example, in the social orienting task, the original stimuli, such as faces and houses, were preserved but enhanced with animations, including a swirling vortex transition and nature, without altering the essence of the stimuli.
- Visual interest: Vibrant colors, dynamic animations, and visually appealing transitions were introduced to maintain children’s attention throughout the tasks.
- Audio: Sound effects and music were added to create a more engaging experience. The sounds were selected to complement the visuals—for example, a chirping sound when a bird appeared on screen.
- Fidelity-engagement compromise: When adding game elements to a cognitive test there is always a trade-off between game elements and the core task [41]. In this study, we made an effort to maintain a balance between making the tasks engaging and preserving the original test’s fidelity. While some tasks, such as the social orienting task, retained their original structure with some adjustments, others, such as the attention task, underwent larger changes to introduce a storytelling element while preserving the main objectives.

**Figure 2:**
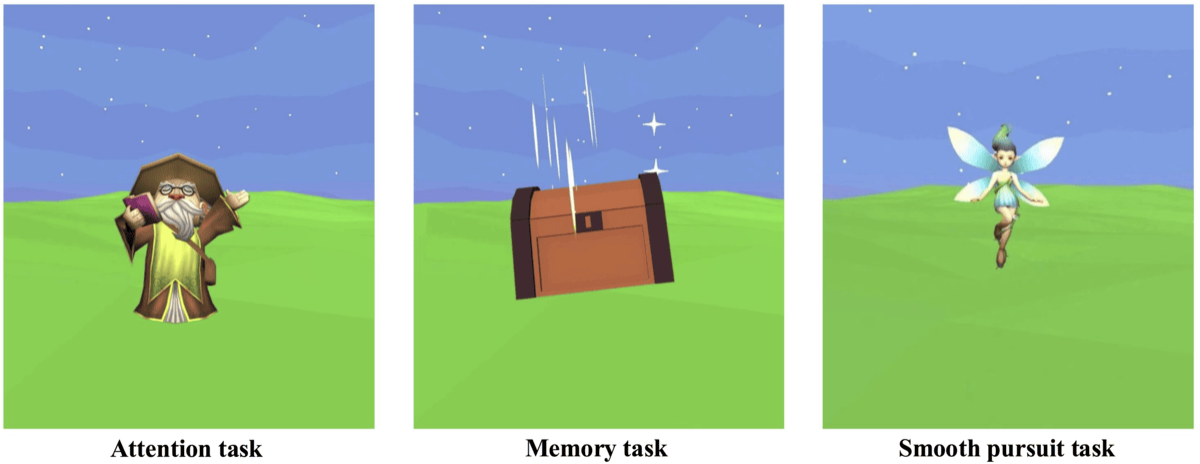
Examples of visual elements in the cartoon version. The wizard casts a spell in the attention task (left), a treasure chest with visual effects is shown in the memory task (center), and a fairy stimulus is presented for the smooth pursuit task (right). A common background is shown behind the objects across all tasks.

This process resulted in several differences from the standard version. The black background of the standard test was replaced with a computer-generated image of a grass field under a twilight sky, with a dark blue sky and stars. This background was consistent across all tasks (Figure 2). While the smiley face in the standard test serves as the initial fixation for all tasks, in the cartoon version, each task has a different target depending on how it is framed. All stimuli in this test are copyright-free Unity models, and all timings from the standard test were maintained; however, due to animations and transitions, the tasks are uniformly longer.

**Figure 3:**
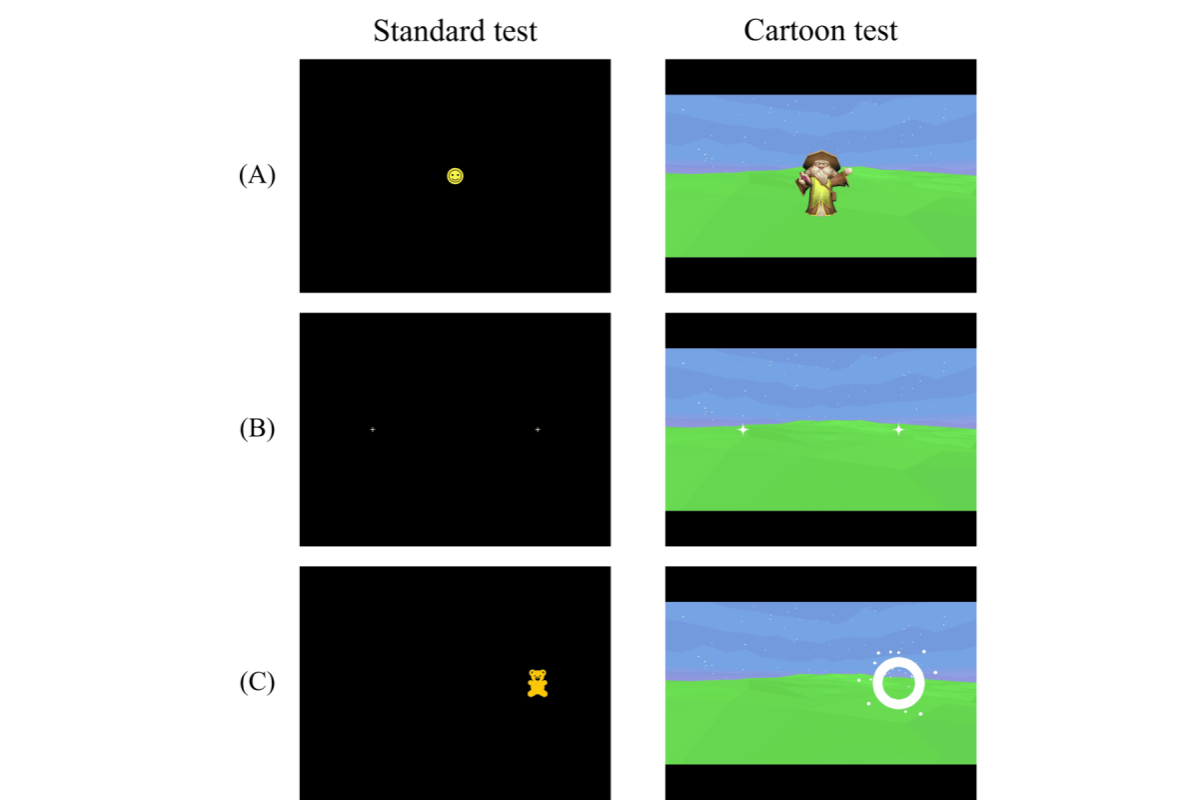
Comparison of the two versions of the attention task: the standard version (left) and the cartoon version (right). (A) Initial fixation (smiley face or wizard), (B) cues (crosses or sparkles), and (C) target (animal icon or fireworks).

The tasks used in the cartoon version were the same as those in the standard version. These 4 task types are described below, along with the differences introduced in the cartoon version. The differences are also summarized in Table 1, and a side-by-side comparison of the attention task is shown in Figure 3. . A video example of the tests is available in Multimedia Appendices 1 and 2.

**Table 1:** Summary of the differences between the two versions of the test.

Element			Standard test		Cartoon test
**Smooth pursuit task**					
	Target	Yellow smiley face		A bird, a dragon, and a witch (horizontal) and a fairy and a slime (vertical)	
**Attention task**					
	Fixation	Yellow smiley face		A magician	
	Cues	White crosses		Small white sparkle	
	Target	Colored animal graphic (dog, cat, etc)		Firework explosion	
**Memory task**					
	Fixation	Yellow smiley face		Shaking treasure chest	
	Pictures (color modality)	Colored circles		Colored spheres	
	Pictures (shape modality)	2D shapes (circle, square, and diamond)		3D shapes (sphere, cube, and diamond)	
	Pictures (face modality)	Photographs		Photographs in a 3D frame	
**Social orienting task**					
	Fixation	Yellow smiley face		Purple vortex	
	Pictures	Photographs		Photographs in a 3D frame	

Smooth pursuit task: In the standard version, a yellow smiley face moves smoothly across the screen in a sinusoidal pattern. There are 4 variants of the task, defined by two speeds along each of the two axes (horizontal and vertical). The periods used are 1600 ms (0.625 Hz) and 2400 ms (≈0.4 Hz). All variants last 7.5 seconds, plus 1.5 seconds for initial fixation with the smiley face. The design of this task was inspired by previous studies using smooth pursuit [4,23]. In the cartoon version, in place of the smiley face, different stimuli move on the screen. The movement is again an oscillation with the same orientation, speed, and amplitude. Before moving, the stimulus remains at the center of the screen for 1.5 seconds to induce fixation. The models used are a bird, a witch, and a dragon for the horizontal variant, and a fairy and a bouncing slime for the vertical one. All of them can be considered characters (no inanimate subjects) to increase engagement and motivate their movements. As they move, a sound is played depending on the model (eg, chirping for the bird and a bell jingle for the fairy). The task is 1 second longer due to a small break before the appearance of the next stimulus (10 seconds).

Memory task: The task starts by showing two pictures for 3 seconds (habituation phase), followed by a blank screen for 0.5 seconds, and then two pictures are displayed for 4 seconds, with one of them replaced by a new image (novelty phase). There are 2 variants of this task: in variant A, based on the work of Ross-Sheehy et al [22], the two initial pictures are different, whereas in the classical visual pair comparison (variant B) [42], the two initial pictures are the same. Furthermore, each variant is presented in 3 distinct modalities, depending on the pictures shown. These include circles of different colors (color modality), different simple shapes (shape modality), and pictures of human faces (face modality). The colors used are red, yellow, and green, while the available shapes are a square, a diamond (a square rotated by 90 degrees), and a circle. The face pictures used in the test are from the London Faces Dataset [43]. In the cartoon version, the initial fixation target is a treasure chest that falls from the top of the screen and stops in the center. The chest then shakes, accompanied by a suitable sound effect, then explodes, and two objects appear in place of the two pictures. Then, after the habituation phase, the two objects leave the field of view by moving off the screen from the bottom. After a short delay, the objects move upward and return to their previous positions, with one of them changed. As in the standard version, the task has 3 modalities: colored spheres (color), different 3D shapes (shape), and framed pictures of faces (face). Due to the animations (eg, treasure chest appearing, objects moving) the task is 3 seconds longer than its standard counterpart, with a total duration of 12 seconds.

Attention task: The task is inspired by the “Infant Orienting With Attention” paradigm [26] and is composed of 4 modalities. In each modality, after the first fixation at the center (the yellow smiley face), little white crosses, called cues, appear to one side or both sides of the screen. The cues stay on the screen for 0.1 seconds with 0.15 seconds blank screens before and after. A sound accompanies the cues. Subsequently, a target (a brightly colored animal graphic, such as a dog or a cat) appears on one side of the screen for a duration of 1 second, and the participant is expected to fixate on this target. The 4 modalities differ on the position of the cues: the valid modality has the cue on the same side where the target will appear, the invalid one has the cue on the opposite position, the double modality has cues on both sides, and finally there is a baseline modality without cues. The 4 modalities are presented in a different order in each repetition. The duration of each modality is 2.9 seconds, resulting in a total duration of the task of 11.6 seconds. In the cartoon version, we framed the entire task to give it a simple story: a wizard tries to cast a magic spell with varying results. The spell is supposed to create a small sparkle (the cue) and then a firework explosion (the target) in the same position, which would correspond to the valid modality in the standard task. As such, depending on the modality, the casting is framed as successful (valid modality), unsuccessful (invalid), or partially successful (baseline, double). All 4 modalities follow the same structure. The wizard first appears at the center of the screen in a puff of smoke, before casting the spell with a short animation; this first phase corresponds to the initial fixation. The wizard then disappears in another puff of smoke and a white sparkle(s) (not animated) appears as the cue(s), followed by an animated firework explosion as the target. Then the wizard reappears, and a different animation is played depending on how successful his spell was: a happy clapping (successful), shamefully hanging his head (unsuccessful), or an unsure humming (partially successful). The addition of these storytelling elements resulted in a significantly increased time: the total task lasts 37 seconds, compared to the 11.6 seconds of the standard version.

Social orienting task: The task follows a paradigm from Telford et al [6]. After the smiley face at the beginning, the participant is shown two pictures, each filling half the screen, one of a house and the other of a human face taken from the London Faces Dataset [43]. The pictures of faces all look forward with a neutral expression and were chosen to maintain an approximate balance of gender and ethnicity across the 4 repetitions. The two pictures stay on screen for 5 seconds; thus, the total duration of the task is 6.5 seconds. In the cartoon version, at the beginning of the task, a bright purple vortex is shown at the center of the screen to attract the participant’s attention, and the two framed pictures appear as if ejected from the vortex, showing the same pictures used in the standard version. Due to the animation of the vortex appearing, disappearing, and the pictures exiting the vortex, the task is slightly longer than the standard version, lasting 8 seconds.

### Recruitment

Participants in this study were recruited between February 2024 and February 2025 from children undergoing periodic checks at the Centre for Developmental Disabilities, University Hospitals Leuven, Belgium. Inclusion criteria were age 4-6 years and born preterm (gestational age <32 weeks, birth weight <1500 g, or both). Children with severe visual impairments (eg, acuity <0.2 on a decimal scale), behavioral problems, or any other impairment that might influence the results of the measurements were excluded from the study.

A total of 26 children (mean age 60.2, SD 2.62 months) were recruited for the study, all of whom were born preterm (gestational age <32 weeks). Among these, one left during the break and was thus unable to have a recording for the second test. No test was interrupted due to unrelated conditions unrelated to the participants, such as malfunctions or a request from the parents. As such, we analyzed data from 25 children, of whom 15 (60%) were male and the remaining female. The median birth weight was 1440 (IQR 1220-1580) g, and the median gestational age was 31 (IQR 30-32) weeks. Among the participants, 12 were shown the cartoon version first (5 females, 7 males) and 13 the standard version first (5 females, 8 males).

### Data Gathering Procedure

To maximize the effectiveness of the recordings while minimizing distractions, we followed the guidelines outlined by Duchowski [44] when creating the setup. Participants were instructed to sit on a comfortable chair positioned 60 cm from a 21-inch computer screen with a mounted Tobii Pro Nano eye-tracker (60 Hz). The child was separated from the rest of the room using a white folding screen, and a second white screen was positioned behind the computer screen. Behind the two screens, an operator could visualize in real time where the participant was looking on the screen and, if necessary, stop the test. The two test variants were then played sequentially, with a 10-minute break in between; the order of the variants was alternated across participants. During the break, children were encouraged to play with their parents and relax.

The operator was instructed to let the test play to its conclusion and interrupt it only if: (1) the child communicated verbally that they wanted to stop, (2) the child was crying, fussy, or unable to continue, (3) the child got up from the chair and walked out of the test area, and (4) the child interfered with the setup of the test (eg, pushed either of the folding screens causing them to fall). All these conditions were interpreted as a definitive loss of engagement from the participant. In addition, the test could also be interrupted due to software or hardware malfunction or after a request from the child’s parents.

After both tests were concluded, the operator asked the participant which version they preferred with the question, “Which video did you like the most?” If the participant preferred the standard version, the operator would ask them the reason.

### Statistical Analysis

#### Measures

For each participant, the recorded gaze trajectories were divided into the 48 tasks composing each test variant. Next, we computed the LDI, a quantity based on robustness, on a task-by-task basis. Robustness is a measure of data quality for eye-tracking data [27,40], which encodes the tendency of the device to incorrectly record a sample and is calculated by dividing the number of nonmissing values by the total number of samples. Robustness offers an upper bound to the amount of data that can be parsed and analyzed in any subsequent study. In this study, we took inspiration from this measure to define the LDI as the ratio between gaze points that were either (1) missing or (2) not positioned inside the screen boundaries, and the total number of samples. Moreover, small gaps in valid samples (ie, <150 ms) were ignored when computing this quantity, as they were probably due to blinking, which represents an unavoidable data loss and can be imputed. An LDI of 0 means no data loss, while a value of 1 signifies that the entire task is unusable; small values are therefore preferable and, depending on the stimuli, tasks with an LDI value above a certain threshold might be discarded [4]. From the LDI value, it is possible to obtain the total time of disruption by multiplying it with task duration; in this study, however, we only used standard LDI to maintain normalization across tasks. The main differences between robustness and LDI are that (1) most disruption due to the device (usually <150 ms) and blinks are ignored by the LDI because they are unavoidable, and (2) gaze positions that are detected but not usable (outside the screen) are counted. These differences result in a slightly different feature that is less about the eye-tracking device and more about the measure of the avoidable disruption caused by the participant. For example, a participant that continuously looks away from the screen because they are distracted will have a higher LDI than a participant who correctly fixates on the stimuli. As such, the LDI is both an upper bound on unusable data and an indication of engagement in the test. The latter interpretation must be considered with caution, since a participant with a perfect LDI of 0 might not be following the tasks and may be completely uninterested, while still looking at the screen. That is, the LDI contains no indication of task performance. Our main motivation for choosing LDI in place of robustness or another feature describing engagement is that LDI is a general measurement that can be computed for all kinds of eye-tracking tasks, in contrast with other outcomes that are specific to certain kinds of tasks (eg, the number of fixations or saccade length depends heavily on the stimulus chosen). Thus, it can be compared across different tasks and potentially generalized to other kinds of tests. Moreover, it provides an indication of both engagement and data quality, as a bound to recording time that can be analyzed. If a certain task is missing from the test, for example, because the operator interrupted the test, the LDI is set to 1, signifying no data available.

Once we defined LDI as a measure of data quality and engagement, we applied two approaches to compare the standard and cartoon variants of the test.

#### Experiment 1: Direct Comparison of LDI and Evolution in Time

We directly compared LDI values between the two tests to show that the cartoon test had significantly lower LDI. The comparison was done by dividing the complete test into 4 repetitions of 12 tasks each. Then, for each repetition, the average LDI over all 12 tasks was computed by weighting each task by its respective duration. The comparison consisted of four 1-tailed Mann-Whitney *U* tests to find evidence that the cartoon test had a lower LDI. We chose the *U* test because LDI is bounded between 0 and 1 and therefore nonnormally distributed.

In addition, we examined the evolution of the LDI during the test and visually investigated its general trend in both versions.

For experiment 1, we set the significance level to α=.05 and applied a Bonferroni correction (N_test_=4) to account for repeated statistical tests (equivalent significance α_Bonf_=α/N_test_=.0125).

#### Experiment 2: Admissible Tasks and Test Length

As a second experiment, we investigated the expected length of the two tests if we were to introduce a stopping criterion based on LDI in a simulated clinical setting. In general, there are two broad categories of testing procedures: a static test, which is kept the same for all participants and has a fixed length, and a dynamic test, which is adapted to the participant and can have a variable length. In this study, a dynamic test is defined as a testing procedure in which we can choose, based on real-time measures, which task to present to the participant. Small LDI values in a static test will lead to fewer repetitions being discarded in the cartoon version, which allows features to be aggregated to obtain more stable outcome measurements. In the case of a dynamic test with a stopping criterion based on lower LDI, the cartoon test will lead to shorter tests on average.

To show that a lower LDI results in a shorter testing time, we simulated a dynamic testing procedure by introducing a plausible stopping criterion for our test. If we were to use a similar criterion in a comparable population, testing time between the two versions would be comparable, and the use of the cartoon version would lead to an increase in correctly completed tests. To simulate a dynamic stopping criterion, we used the same dataset and artificially censored data after the simulated stopping time. The criterion used in this study to decide if a task should be discarded is based exclusively on lost data. Additional motivation to discard tasks could be low precision or accuracy [40], but precision and accuracy thresholds usually depend on the specific quantity measured (eg, smaller areas of interest necessitate higher precision). As such, to keep our considerations as general as possible and in line with the preceding considerations, we did not include them in the analysis, focusing on LDI. Specifically, we considered a task admissible if its LDI was below a certain threshold τ. This criterion for discarding sections of recordings is not new in eye-tracking applications, for example, Gredebäck et al [4] use τ=0.2 in their smooth pursuit study.

The stopping criterion chosen in this study was intended to be as general as possible and rests on the reasonable assumption that a certain number of tasks is necessary to reach a diagnosis. Thus, the test was run normally and could be stopped prematurely once we record M different admissible (not discarded) tasks, where M ranged between 1 and 12 (in our analysis M=12; thus, all tasks needed to be admissible to obtain a complete test). If the fourth repetition concluded without reaching the desired number, the test was deemed incomplete. It is important to note that, in a real dynamic setting, it would be possible to select the order of the tasks and present only the missing ones to the participant. This approach has some drawbacks: for example, it would lead to test recordings with tasks in different orders, and therefore researchers would need to consider order effects during the trial and analysis. In addition, simulating this approach with our dataset would introduce distortion in the results, since tasks were always presented in the same order. For these reasons, in our simulation we assumed the tasks were always presented in the same order, and every time a certain task was not admissible, it was necessary to run a new repetition.

**Figure 4:**
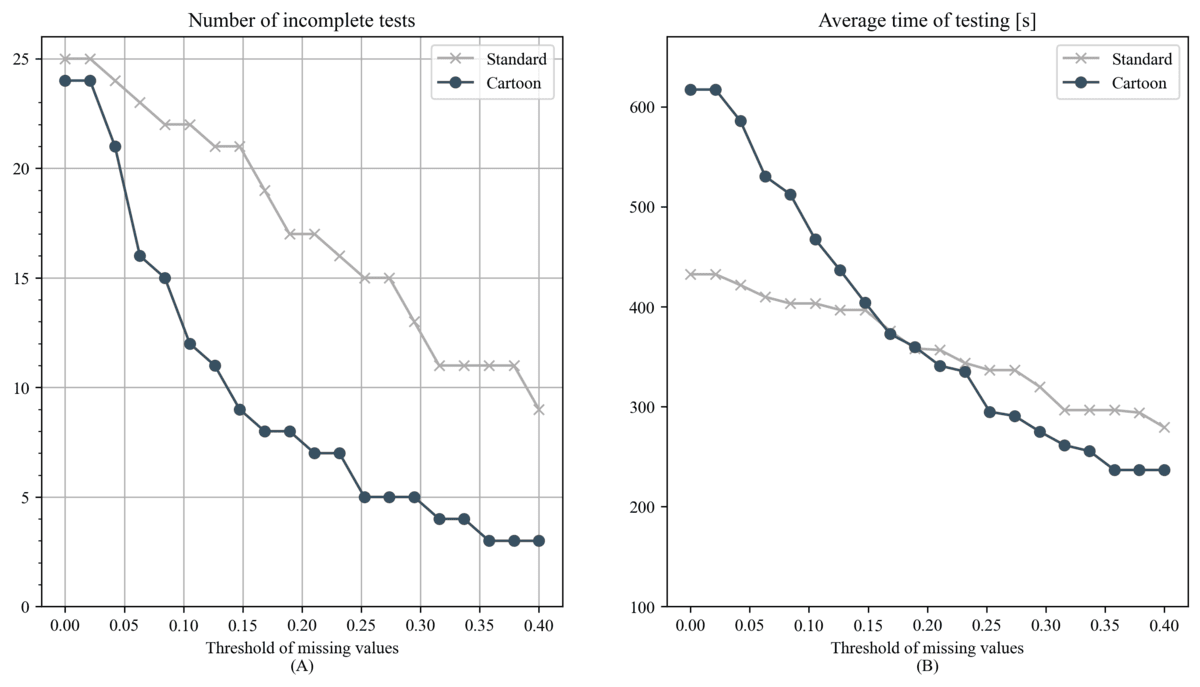
(A) Number of participants who would have successfully completed the test for each plotted threshold τ. (B) Average total duration of the simulated dynamic test with the stopping criterion for each threshold τ. In panel (B) for τ=0 (no missing value tolerated), both tests run their entire course, and the cartoon test is longer.

Since test length and number of admissible tasks depend on the threshold τ, we obtained a curve for each test version (τ→T_duration_), which described test length when varying the threshold. A numerical comparison could be done by fixing a certain τ—that is a point comparison (in this study, we chose the τ=0.2, inspired by previous work [4])—or by computing the area under the curve (AUC) for each participant. It is worth noting that this AUC referred to the curve shown in Figure 4 and should not be confused with the area under the “receiver operator characteristic” curve or the same term used in pharmacokinetics. For both point comparisons and AUC, we used a 1-tailed Mann-Whitney *U* test (significance level α=.10) to determine whether there was a significant difference in test length between the standard and cartoon tests when using a dynamic stopping criterion (with the alternative hypothesis that the cartoon test was longer).

### Ethical Considerations

This study was conducted with the approval of the Ethical Committee KU Leuven/UZ Leuven (EC number S68019) and on the basis of previous informed consent by the participants’ parents or their legally authorized representatives. Eye-tracking data are naturally anonymous, as it is impossible to clearly identify a person from gaze movements alone. Clinical data were partially anonymized by removing names and surnames. All personal data were treated as confidential by the researchers and not shared with anyone external to this study.

## Results

### Experiment 1: Direct Comparison of LDI and Evolution in Time

**Figure 5:**
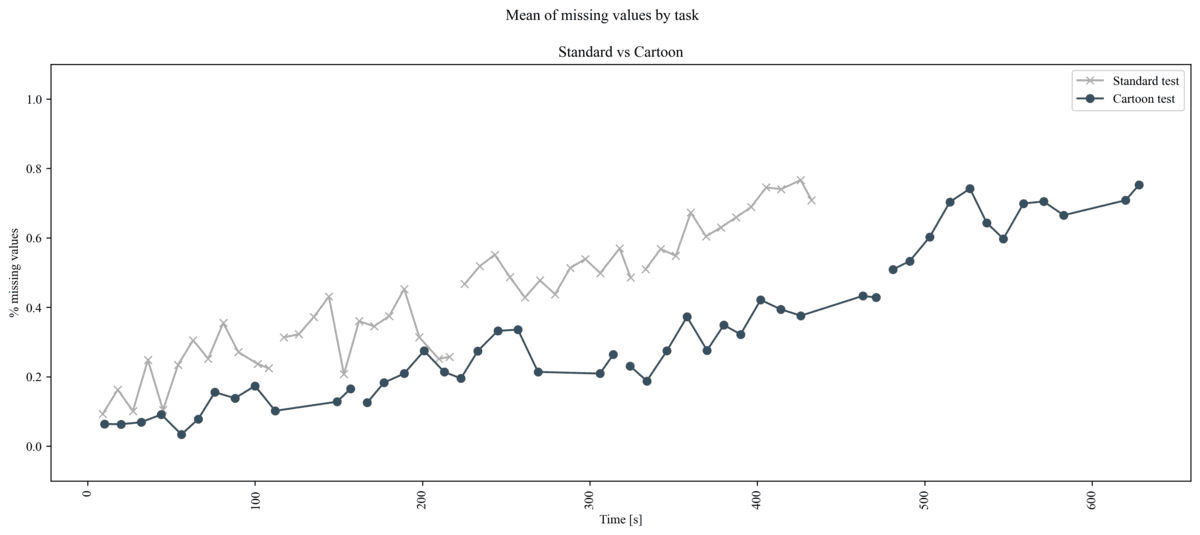
Average lost data index over time for the two versions of the test. The vertical axis represents the lost data index by task, and the horizontal axis represents time. The cartoon test is longer. Repetitions are separated by gaps in the lines.

**Figure 6:**
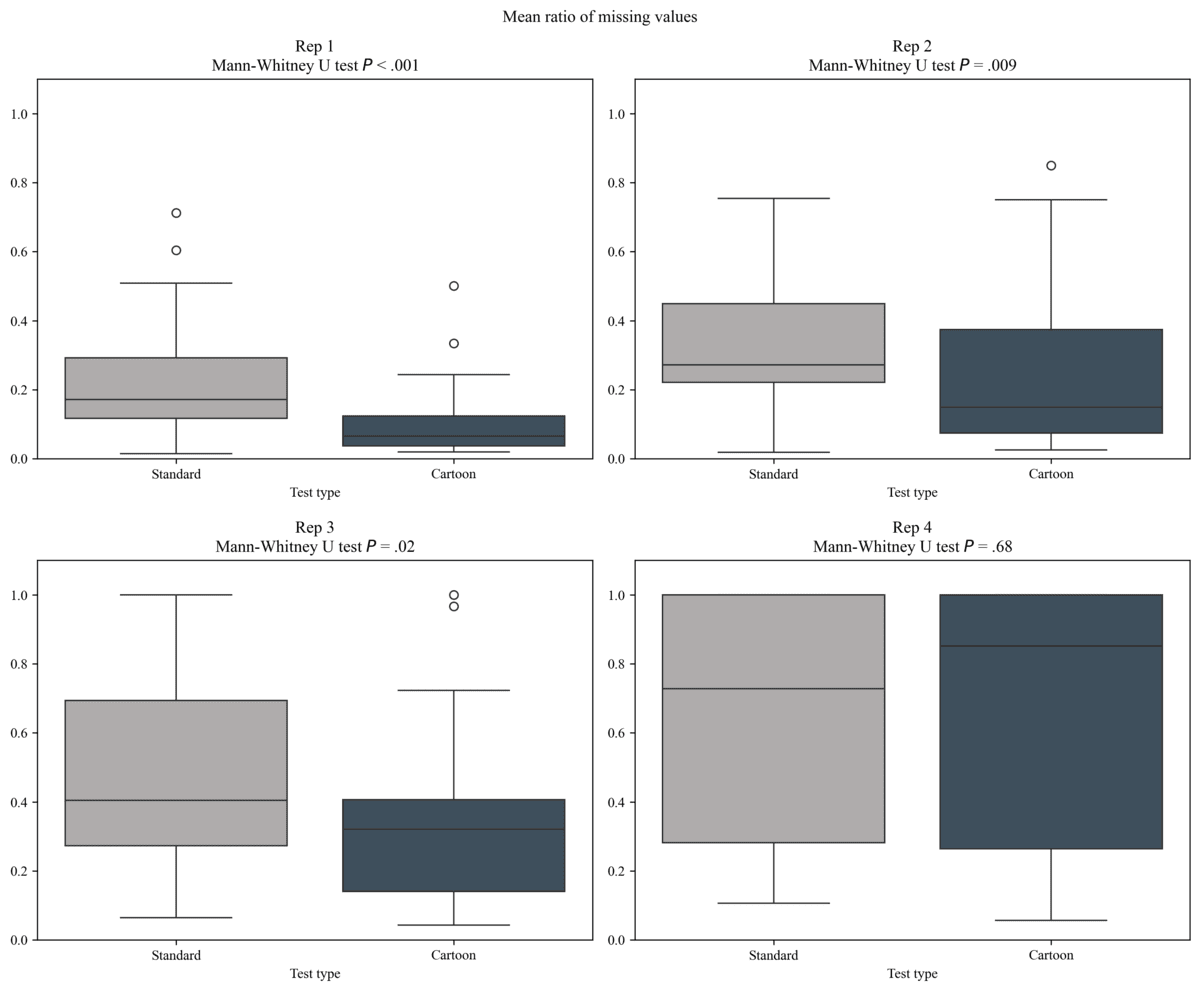
Aggregated lost data index values by repetition with *P* values from the 1-tailed Mann-Whitney univariate *U* test.

After computing the LDI by task, qualitative inspection of its trend over time (Figure 5) shows an approximately linear effect of testing time on the average engagement of the participants. Aggregating the values reveals evidence that the LDI of the entire test is lower in the cartoon version, but not significantly (*P*=.05, Mann-Whitney *U* test, Table 2). This is because the effect is not uniformly distributed across the entire test: the initial repetitions show a larger gap in LDI values between the two versions. In the first repetition, the cartoon version retains a significantly larger percentage of data (*P*<.001; median cartoon LDI 0.11, IQR 0.04-0.12 vs median standard LDI 0.22, IQR 0.12-0.29). This effect decreases over the course of the test, and in the last repetition the LDI is approximately the same between the two versions (*P*=.68). Figure 6 shows the distribution for each repetition, together with the respective *P* values.

**Figure 7:**
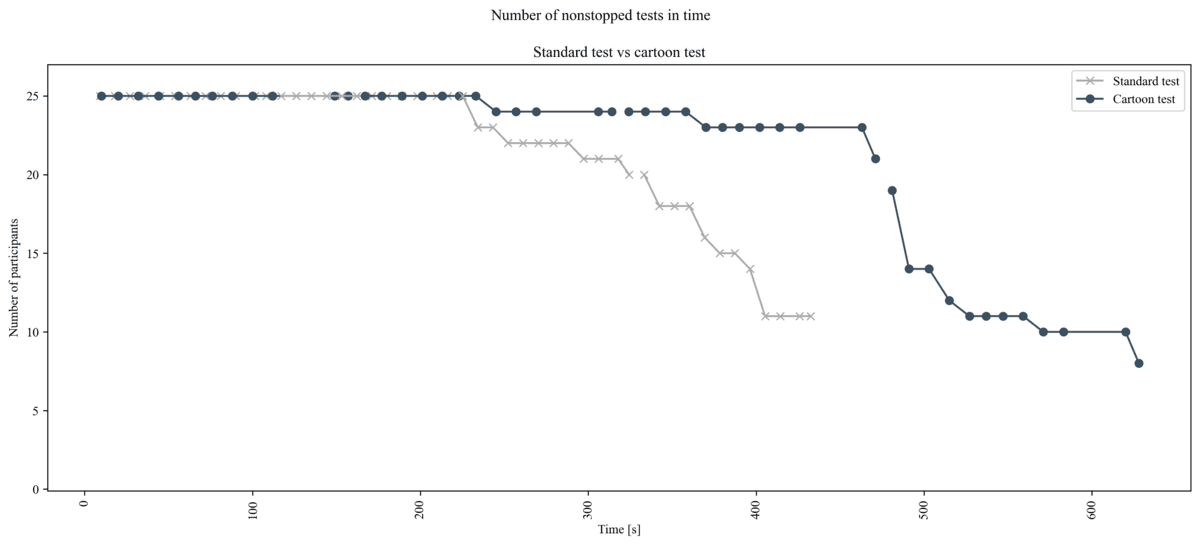
Number of uninterrupted tests over time for the two versions of the test. The vertical axis indicates the total number of participants who had not interrupted the test at a given time point.

We propose two explanations of the observed phenomenon: first, because of the increased test length, the fourth repetition of the cartoon version starts at 471 seconds, more than two minutes after the start of the fourth repetition in the standard version (325 seconds). Thus, it is possible that, due to the effect of testing time on LDI (Figure 5), we have reached the limit of engagement for the participant. Second, this effect might be caused by a form of survival bias. Indeed, by inspecting when tests were interrupted (Figure 7), we observe that the cartoon test is usually stopped later, with only 16% (4/25) of participants interrupted before 432 seconds in the cartoon version versus 56% (14/25) in the standard version, but both of them are stopped in the third and fourth repetition in comparable numbers. Therefore, in the fourth repetition the participants that are still being tested are probably the more attentive ones, independently of the variants shown, and so data loss is comparable.

**Table 2:** Summary of results. For each hypothesis test, the *P* value and average values of the quantity compared are reported. Average values are reported as mean (standard deviation). Separately, the first quartile and third quartile are reported. All tests were performed using the 1-tailed Mann-Whitney U test.

Test		*P* value	Value standard, mean (SD)	Value standard, IQR	Value cartoon, mean (SD)	Value cartoon, IQR
**Experiment 1**						
	Complete test (LDI^a^)	.05	0.43 (0.19)	0.26-0.54	0.33 (0.19)	0.19-0.45
	Repetition 1 (LDI)	<.001	0.22 (0.17)	0.12-0.29	0.11 (0.11)	0.04-0.12
	Repetition 2 (LDI)	.01	0.33 (0.17)	0.22-0.45	0.24 (0.22)	0.07-0.37
	Repetition 3 (LDI)	.02	0.50 (0.27)	0.27-0.69	0.34 (0.25)	0.14-0.41
	Repetition 4 (LDI)	.68	0.65 (0.32)	0.28-1.00	0.66 (0.37)	0.26-1.00
**Experiment 2**						
	Test time (τ=0.2, in seconds)	.21	357 (117)	270-432	360 (201)	157-628
	Test time (AUC^b^)	.50	75.6 (17.6)	60.3-91.0	80.7 (27.4)	56.0-99.6

^a^LDI: lost data index.

^b^AUC: area under the curve.

These results are also reflected in the stated preferences of the participants: of the 25 children who have completed both tests, a total of 14 answered the follow-up question about which test they preferred. Out of the 14 participants, 11 (79%) showed a preference for the cartoon version over the standard version, while 2 (14%) preferred the standard version, and 1 (7%) indicated that they did not like either version. The 2 participants who preferred the standard version provided distinct reasons: one participant was scared by the wizard of the cartoon version (Figure 2), while the other liked the standard version because it was shorter. Figure 6 shows aggregated LDI values by repetition, with *P* values of the 1-tailed univariate *U* test.

### Experiment 2: Admissible Tasks and Test Length

**Figure 8:**
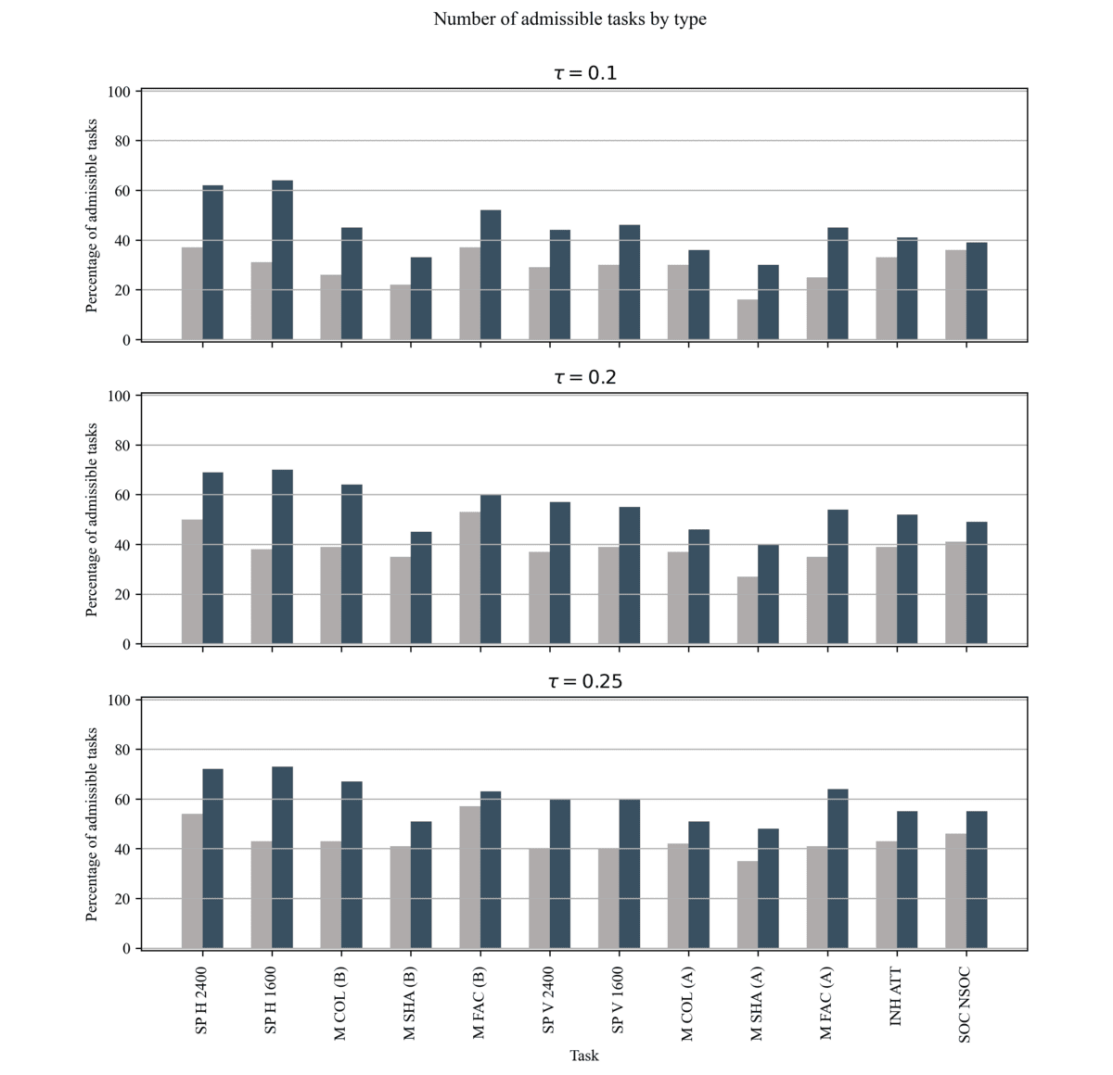
Percentage of total admissible tasks for τ=0.1, τ=0.2, and τ=0.25, divided by task type. A task is considered admissible if the lost data index was ≤τ. INH ATT: attention task; M COL (A/B): memory task-color (variant A/B); M FAC (A/B): memory task-face (variant A/B); M SHA (A/B): memory task-shape (variant A/B); SOC NSOC: social orienting task; SP H 1600: smooth pursuit task horizontal-fast; SP H 2400: smooth pursuit task horizontal-slow; SP V 1600: smooth pursuit task vertical-fast; SP V 2400: smooth pursuit task vertical-slow.

Given the results of experiment 1 (ie, smaller LDI in the initial repetitions of the cartoon version), a practical consequence is that fewer tasks would be discarded due to data loss. This would result in either more robust measurements when using a static test or a shorter procedure when using a dynamic test. Figure 8 illustrates this effect: for 3 threshold value (τ=0.1, τ=0.2, and τ=0.25) the total number of admissible tasks is consistently higher in the cartoon test. This is expected given the lower LDI demonstrated in the previous section and should not be interpreted as additional evidence. Instead, it serves as an example of expected behavior in a clinical setting. Additionally, Figure 4 shows how the number of incomplete tests and the average testing time vary as the parameter τ is changed. Then we conducted a point comparison using a value of τ=0.2 and another comparison by computing the AUC for each participant. The point comparison shows that at τ=0.2, 68% (17/25) of participants had incomplete standard tests, compared with 32% (8/25) for the cartoon test. In addition, there is no strong evidence that testing time is higher for the cartoon version (*P*=.21 *U* test). When comparing AUCs, the situation is analogous, and a Mann-Whitney test shows that there is no strong evidence that the cartoon test has a higher AUC compared with the standard test (*P*=.50). Table 2 provides a full exposition of the results.

## Discussion

### Principal Results

In this study, we investigate the effect of gamification on participants’ engagement during an eye-tracking test battery in a cohort of 5-year-old children born preterm. This age group was selected based on observations from previous studies [14,36], in which we identified challenges in capturing the attention of older children compared with infants.

The core result of this study is that, compared with a standard testing procedure, the cartoon version better maintained participant’s attention on the screen in the considered population. The first 3 repetitions of the tasks displayed a lower LDI in the cartoon version, indicating that participants focused on the screen more often and were less frequently distracted. This result is supported by the subjective feedback of the participants: among those who expressed a preference, the majority (11/14, 79%) preferred the cartoon test. Among the 2 (2/14, 14%) that preferred the standard test, one was scared by an element, and the other felt that the test was too long. This finding underscores the importance of selecting appropriate gamified stimuli and suggests that a dynamic stopping criterion might enhance the experience.

Another observation of this study is the decline in engagement at the fourth repetition across both the cartoon test and standard assessment. This consistent trend suggests that the repetitiveness of the stimuli plays a significant role in reduced motivation, and that gamification cannot fully compensate for this decline. The phenomenon can be attributed to various factors, including boredom or habituation to stimuli, all of which are prevalent in pediatric populations with shorter attention spans [15]. As such, it is essential to ensure high-quality recordings during the initial repetitions of the stimuli. The cartoon test, despite its engaging nature, is not immune to these effects. However, its ability to mitigate the impact of test duration relative to the standard test is noteworthy, with a higher retention rate (21/25, 84% vs 15/25, 60%, an increase of 40%).

By incorporating visually stimulating elements, the cartoon test increases task engagement and reduces the monotony associated with repeated exposure to similar stimuli. Moreover, the capacity for longer testing durations and the inclusion of more admissible tasks, due to a lower LDI, address two limitations of traditional assessment methods. By sustaining engagement through visually appealing and dynamic content, the cartoon test enables researchers to gather a more extensive dataset within a single session. This can be clearly seen in the results summarized in Figure 4, where the two tests, if dynamic stopping is used, reach a similar duration, indicating that with the cartoon test fewer repetitions are sufficient to gather enough data for analysis. Indeed, with the stopping criterion described and τ=0.2, about 40% (10/25) of the cartoon tests required only 1 repetition to obtain enough admissible tasks, compared with 9% (2/25) of the standard tests, thereby minimizing the disruptive effect caused by a large number of repetitions. These results show that gamification of eye-tracking test batteries for 5-year-old children born preterm is a promising research direction to increase data quality and engagement, without necessarily adding to the duration of the procedure.

It is also important to note that the effect of gamification on task performance is outside the scope of this study. In contrast with previous studies [16,18,21], we focused exclusively on the engagement and data quality aspects of gamification.

### Comparison With Previous Work

Our results appear to be in contrast with a previous study by Scharinger et al [34], in which they used pupil diameter and fixation duration to measure engagement in a gamified version of the N-block test. By comparing with the standard version of the exercise, they did not find a significant effect of gamification on the measured outcomes. This lack of a difference between the two versions can be explained by the age of their sample, which was composed of university students, or by the choice of measured outcomes: LDI in our case and pupil diameter in theirs. It is worth noting that, even without a measurable difference in gaze behavior, the participants found the gamified version subjectively more interesting. In contrast, our results agree with a similar comparison conducted by Friehs et al [21], in which a cohort of university-aged participants displayed similar performance and increased enjoyment with the gamified paradigm. The main difference with previous studies is that, in our case, the age of the participants makes it challenging to use a questionnaire to measure engagement with the test. Consequently, we have focused on the eye-tracking measures such as LDI to compare the two versions, in addition to short qualitative feedback from the participants. Previous research by Wiley et al [16] also highlights that the increase in engagement brought by gamification can increase consistency in performance of the stop-signal task between multiple tests in an adult population. This would be in accordance with our own results, since low LDI values and a high number of admissible tasks are linked to more stable measurements. However, again, the key dissimilarity is the age difference in the participants. Gallaghar et al [18] have tried to extend the results of Wiley et al [16] to younger participants (aged 8-12 years) with attention deficit hyperactivity disorder, receiving positive feedback, although a direct comparison with the standard version of the task was not included.

### Limitations

Finally, while the cartoon version demonstrates considerable promise, this study is not without limitations. First, we had a small sample size, which restricts the generalizability of findings and is the reason we focused on a single quantity of interest and did not take into account the effects of specific task types. Second, our approach of considering only the LDI, while increasing the scope and generalizability of our results, could be complemented by other measures such as pupil size [34]. Finally, while some of these results might translate to different populations, as shown in other studies [16,18,21], the test we used was designed specifically for young children, and we cannot assume that similar results would be found in adults.

### Future Work

The results of this study suggest that future work in the development of cartoon versions of existing eye-tracking paradigms could be game-changing when testing young children, who are otherwise difficult to keep engaged. Future work should focus on further analysis including pupil size [34] and task performance [16]. Moreover, in this study we did not consider carry-over effects due to task order, and this would be a crucial step in creating a dynamic version of the test, where gamification could be used to shorten the procedure.

### Conclusions

In this paper we directly compared two versions of the same eye-tracking test battery, a standard version, and a gamified cartoon version. We have shown that within a group of preterm born five-year-old participants, the cartoon version is correlated with a lower level of data loss and distraction by the participant. This difference is especially present in the first repetitions of the tasks, and its decline during the test might be due to the repetitiveness of the stimuli. Nonetheless, due to the lower data loss caused by gamification, we can expect not only to improve data quality in the recordings but, by implementing dynamical stopping criteria, shorten the testing procedure. Our results provide practical guidelines to gamifying eye-tracking tests by offering a direct comparison and, by implementing the recommendations outlined in the discussion, researchers can refine the design of serious games and maximize their potential as a reliable and effective tool in pediatric cognitive research.

## Appendix

Multimedia Appendix 1 Example of some of the tasks in the standard test. Tasks: Smooth pursuit task (horizontal), Memory task (colour modality, variant B), Memory task (face modality, variant B), Attention task (valid modality only), Social orienting task.

Multimedia Appendix 2 Example of some of the tasks in the cartoon test. Tasks: Smooth pursuit task (horizontal, bird), Memory task (colour modality, variant A), Memory task (face modality, variant A), Attention task (valid modality only), Social orienting task.

## Abbreviations

AUC: area under the curve
LDI: lost data index

## Data Availability

The datasets generated or analyzed during this study are available from the corresponding author upon reasonable request.
